# Knowledge and attitudes toward antimicrobial use, resistance, and stewardship among final-year veterinary students: a multinational cross-sectional survey

**DOI:** 10.3389/fvets.2026.1957292

**Published:** 2026-10-06

**Authors:** Mahmoud Eltholth, Jadesola Juliana Ajao, Shrikrishna Isloor, Ismail Ayoade Odetokun, Ali Raza, Médiha Khamassi Khbou, Dominic Moran, Ilhem Zouyed, Jennifer Cole, Jonathan Rushton, Nagendra R. Hegde, Alison Prendiville, Ghazal Nabil, Uduak Akpabio, Shuaibu Gidado Adamu, Mary Idowu Olasoju, Eman Ahmed Abo Elfadl, Mehmet Cemal Adiguzel, Selim Adewale Alarape, Muhammad Akbar Shahid, Roshan Riaz, Nicola Williams

**Affiliations:** 1 Department of Biological Sciences, Royal Holloway University of London, Egham, United Kingdom; 2 Department of Animal Hygiene and Preventive Medicine, Faculty of Veterinary Medicine, Kafrelsheikh University, Kafrelsheikh, Egypt; 3 Department of Veterinary Microbiology, Veterinary College, Karnataka Veterinary Animal and Fisheries Sciences University, Bengaluru, India; 4 Department of Veterinary Public Health and Preventive Medicine, University of Ilorin, Ilorin, Nigeria; 5 Department of Veterinary Microbiology, Faculty of Veterinary Medicine, Ataturk University, Erzurum, Türkiye; 6 Laboratory of Infectious Animal Diseases, Zoonoses and Sanitary Regulation, National School of Veterinary Medicine of Sidi Thabet, Institution of Agricultural Research and Higher Education, University Manouba, Sidi Thabet, Tunisia; 7 Division of Global Agriculture and Food System, The Royal (Dick) School of Veterinary Studies and the Roslin Institute, Midlothian, United Kingdom; 8 Institute of Veterinary Sciences, University Frères Mentouri Constantine 1, Constantine, Algeria; 9 Department of Geography and Sociology, Royal Holloway University of London, Egham, United Kingdom; 10 GBADs, Roslin Institute, The Royal (Dick) School of Veterinary Studies and The Roslin Institute, Midlothian, United Kingdom; 11 Biotechnology Research Innovation Council, National Institute of Animal Biotechnology, Hyderabad, India; 12 School of Design, LCC, University of the Arts London, London, United Kingdom; 13 Department of Pharmacology, Faculty of Veterinary Medicine, Cairo University, Giza, Egypt; 14 Department of Veterinary Public Health and Preventive Medicine, College of Veterinary Medicine, Michael Okpara University of Agriculture, Umudike, Nigeria; 15 Department of Veterinary Public Health and Preventive Medicine, Faculty of Veterinary Medicine, University of Maiduguri, Maiduguri, Nigeria; 16 Department of Veterinary Public Health and Preventive Medicine, College of Veterinary Medicine, Federal University of Agriculture, Abeokuta, Nigeria; 17 Faculty of Veterinary Medicine, Egyptian Chinese University, Nasr City, Cairo, Egypt; 18 Department of Veterinary Public Health and Preventive Medicine, University of Ibadan, Ibadan, Nigeria; 19 Department of Pathobiology, Faculty of Veterinary Sciences, Bahauddin Zakariya University, Multan, Pakistan; 20 Department of Animal Nutrition and Nutritional Diseases, Faculty of Veterinary Medicine, Kafkas University, Kars, Türkiye; 21 Institute of Infection, Veterinary and Ecological Sciences, University of Liverpool, Neston, United Kingdom

**Keywords:** antimicrobial resistance, antimicrobial stewardship, antimicrobial use, KAP, veterinary students

## Abstract

**Introduction:**

This study assessed final-year veterinary students’ knowledge and attitudes regarding antimicrobial use (AMU), antimicrobial stewardship (AMS), and antimicrobial resistance (AMR).

**Methods:**

Students were invited to complete an online semi-structured questionnaire. A total of 907 participants from 7 countries and 43 veterinary educational establishments (VEEs) completed the questionnaire. Data were analysed using descriptive and inferential statistics. The total knowledge and attitude scores for each student were calculated, and a general average was obtained.

**Results:**

AMR was mostly taught as an addendum under the core subjects of microbiology and pharmacology. Students exhibited low AMS knowledge scores (mean score 32.1 ± 22.7), but higher scores for positive attitudes towards reducing AMR (64.2 ± 25.2). Only 6.6% could name at least one national regulation guiding AMU in their countries, while 30-50% of the students knew that antibiotics were inappropriate for various viral infections. About 42% considered it acceptable to use antimicrobials for growth promotion and prophylaxis. Students exhibited positive attitudes towards using antimicrobial susceptibility testing, and 50.1% considered improved veterinary education very important for reducing AMR. Multivariate analyses determined that students’ countries of origin were significantly associated with their knowledge and attitudes; for example, Nigerian students were more likely to have good knowledge than Egyptian students, (aOR: 2.372, 95% CI:1.085 – 5.551), while Indian students were less likely (aOR: 0.144, 95% CI: 0.063 – 0.343).

**Discussion:**

Greater curricula emphasis should be placed on national and international AMS guidelines. Concepts related to AMR and AMS may be better retained if taught as a standalone module. Further studies should be conducted across countries where students have greater knowledge to evaluate curricula and potentially highlight replicable models for the inclusion of AMR and AMS.

## Highlights

- Most students were not taught AMR as a standalone subject.
- Students exhibited low awareness and knowledge of AMS terminologies.
- Only 6.6% of students could name one national regulation guiding veterinary AMU.
- Students’ knowledge and attitudes significantly varied based on their country.

## 1 Introduction

Antimicrobial resistance (AMR) has emerged as one of the foremost threats to global public health (1, 2). In 2021, 1.14 million human deaths were attributable to bacterial AMR, a number projected to reach 10 million by 2050 without adequate intervention (3, 4). AMR is driven by the misuse and overuse of antimicrobials; however, the uniqueness of AMR as a global challenge lies in its ability to cross human, animal, and environmental sectors (5, 6). Due to this interconnectedness, the United Nations Quadripartite, made up of the World Health Organisation (WHO), World Organisation for Animal Health (WOAH), Food and Agriculture Organisation (FAO), and United Nations Environment Programme (UNEP) has emphasised the need for an integrated ‘One Health approach’ against AMR (6, 7). The UNQ has developed a Global Action Plan on AMR that includes a call for increased stakeholder awareness while optimising antimicrobial use (AMU) across all sectors (White and Hughes, 2019) (8). Yet, a multisectoral approach is impeded in practice by a prioritisation of humans over other sectors in research and development efforts (7, 9). With global veterinary AMU rapidly increasing and projected to increase from 99,502 tonnes in 2020 to 200,235 tonnes by 2030 (9, 10), it is clear that global antimicrobial stewardship will require more support within the animal sector to define and evaluate effective mitigating interventions.

Antimicrobials in the animal sector are used for growth promotion and prophylactic purposes rather than solely as therapeutic agents, and these practices have been reported across low, middle, and high-income countries worldwide for more than 50 years (11, 12). In China, 53.2% of the 29,774.09 tonnes of antimicrobials used in the veterinary sector in 2018 were for growth promotion (11). These practices are thought to significantly contribute to the rise of AMR not only in animals, but also in humans and the environment (12). Thus, recent efforts are being made to increase responsible AMU in animals, while highlighting veterinarians as key stakeholders in this regard (5, 11,13,14). These efforts include guidelines set by the World Organisation of Animal Health (WOAH) for antimicrobial stewardship (AMS) in the veterinary field, including a limitation on antibiotics for metaphylactic, prophylactic and growth promotion purposes and avoiding the use of human antibiotics which have been classified as highest priority clinically important antimicrobials (HP-CIAs) in food-producing animals (13, 15). Table 1 summarises some of the main guidelines for veterinarians.

**Table 1:** Guidelines for responsible use of antimicrobials among veterinarians, summarised from WOAH’s Terrestrial Animal Health Code, 2024 (15).

Category	Guidelines for veterinarians
Prevention	Promote practices such as good biosecurity and vaccination to minimise overall AMU in animals.
	Consider safe and effective alternatives such as vaccines before prescribing antimicrobials.
Treatment	Use antimicrobial agents only for microbial infections and after appropriate diagnosis (pathogen identification and antimicrobial susceptibility testing).
	Veterinarians should dispense antimicrobial agents with consideration of the WOAH List of Antimicrobial Agents of Veterinary Importance (e.g., by avoiding HP-CIAs).
	Veterinarians should avoid the use of broad-spectrum antimicrobials and use narrow-spectrum antimicrobials when possible.
	Veterinarians should adhere to national legislation on acceptable daily limits, maximum residue limits, and withdrawal periods when prescribing antimicrobials for food-producing animals.
Records	Veterinarians should endeavour to maintain strict record-keeping regarding the antimicrobials they dispense, in accordance with national legislation.

AMU, Antimicrobial use; WOAH, World Organisation for Animal Health; HP-CIAs, Highest priority clinically important antimicrobials.

While these guidelines can promote more sustainable AMU in the animal sector, several issues might prevent their execution across countries. These include poor knowledge of AMR and a lack of acknowledgement of veterinarian’s responsibility for reducing AMR, as well as generally low awareness of AMU regulations (16–19). Lower knowledge of AMR regulations among practising veterinarians has been associated with gaps in veterinary curricula (20). In India, an overview of the recent veterinary curriculum noted only scattered inclusion of AMR with no inclusion of withdrawal periods, and other guidelines by WOAH (21). Similarly, in Nigeria, only 27.9% of veterinarians were aware of national antimicrobial regulations, and most of these had gained this information from sources other than their formal veterinary educational establishments (VEEs), including governmental leaflets and the Internet (16).

A key complication in AMS education for veterinarians and veterinary students is the discrepancy between guidelines issued by international bodies (such as WOAH) and the priorities of individual nations (22). In countries where antimicrobial regulations are not strongly enforced by the government, resident veterinarians may also disregard the need for a personal adoption of AMS guidelines (16). However, the level of awareness among individual veterinarians, regardless of national policies, can significantly improve AMU practices, bearing important ramifications for AMS (16, 21). Thus, strengthening veterinary curricula and improving awareness of veterinary students is even more critical.

The UNQ emphasises coordinated responses and educational awareness for veterinarians (6, 23). However, most current studies assessing the knowledge levels of veterinarians and veterinary students are fragmented, focused on singular localities, countries or continents. This study attempts to bridge this gap by administering an online questionnaire across seven countries. We aimed to assess the knowledge and attitudes of final-year veterinary students regarding AMS, AMU, and AMR, and highlight knowledge gaps and possible areas for curricula development.

## 2 Materials and methods

### 2.1 Target population

The target population for this study was final-year veterinary students, particularly those who could be reached through NetVet4SAMU (International Network of Veterinary Education for Sustainable Antimicrobial Use and Tackling Antimicrobial Resistance), an international veterinary network focused on addressing AMR and promoting sustainable AMU. Participants were from 43 VEEs across Algeria, Egypt, India, Nigeria, Pakistan, Tunisia, and Turkey (Supplementary Table 1). Ethical practices, including informed consent, data anonymity, and right to withdraw, were carried out in accordance with the Declaration of Helsinki (24).

### 2.2 Sample size

The initial sample size was determined to be a minimum of 385 participants for the whole aggregated sample using Epitools, based on an assumed 50% awareness of AMU and AMR, a 95% confidence level, 5% desired precision, and an unknown population size.

### 2.3 Questionnaire design

An anonymous online questionnaire was designed based on the objectives of the study by knowledge experts. A group of experts were also recruited to review the questionnaire and assess content validity, relevance and appropriateness. Following this, it was pretested with 10 students to further ensure validity and reliability, with necessary revisions made before full deployment. The questionnaire (Supplementary File 1) included questions to collect the following data: participants’ background and demographics (Section A); knowledge and attitudes regarding AMS and AMU (Section B), and knowledge and attitudes regarding AMR (Section C). Knowledge questions were either binary or open-ended, while attitude questions were on a Likert scale, “not important” to “very important” (1–5). There were 47 knowledge questions and 63 attitude questions.

### 2.4 Data collection and management

The questionnaire was administered online using the Jisc online survey platform. The link to the questionnaire was shared with the participating VEE administrators, who then shared it with their students. Participation by schools and students was completely voluntary. The survey was conducted in English except in Algeria, Tunisia, and Turkey, where students completed it in French and Turkish, respectively. The translation of the questionnaire and responses into French and Turkish was done by native speakers who were also fluent in English. After the questionnaire was completed and submitted, survey data from all countries were exported to a Microsoft Excel Spreadsheet.

### 2.5 Statistical analysis

Statistical analysis was conducted using R and Microsoft Excel. Demographics were summarised as frequencies and percentages. Participants’ knowledge and attitudes were summarised with tables, charts, and graphs. For further analysis, a scoring system was developed. Knowledge questions with binary answers ‘Yes’ and ‘No’ were assigned ‘1’ or ‘0’ accordingly, with reverse scoring applied to negative questions. Open-ended questions assessing definitions were manually reviewed according to the WHO’s and WOAH’s standard definitions to determine correctness, with a scoring of ‘1’ for adequate responses and ‘0’ for incorrect responses. Missing responses were scored as ‘0’, however students who did not give a response to more than 50% of questions in a sub-section were excluded from further calculations. Attitude questions were awarded 1–5 points on a Likert scale; however, reverse and midpoint scoring were applied to appropriate questions. Negative questions included questions related to factors influencing AMU that would promote the development of AMR and was a detriment to AMR, such as product promotions and feed-conversion ratio. Midpoint questions included questions that were determined by the research team as potentially relevant to the development of AMR, including owners’ desire and cost-effectiveness. Missing attitude responses were scored 1, with an exclusion criterion similar to the knowledge section. Total scores for each section and subsection were converted to percentages and summarised with means and medians. To conduct inferential analysis, individual participant scores were grouped into ‘satisfactory’ and ‘unsatisfactory’ with a cut-off of ≥ 70%, consistent with previous KAP studies that have used this as an established threshold (25). This was preferred over Bloom’s 80% threshold due to the binary classification adopted here, which contrasted with Bloom’s three-level classification (26). Pearson’s chi-square test determined bivariate associations at the *p* ≤ 0.20 level. Independent variables with a statistically significant association were entered simultaneously into a multivariate logistic regression model to identify factors associated with knowledge and attitudes. Multicollinearity was assessed using the variance inflation factor, and no evidence of problematic multicollinearity was found (adjusted GVIFs = 1.00–1.03). Model fit was also evaluated using the Hosmer–Lemeshow goodness-of-fit test. Fisher’s exact test was also used to determine associations between knowledge of national regulations and demographic factors.

## 3 Results

### 3.1 Demographics and preliminary information

A total of 907 responses were received from participants in seven countries and 43 VEEs. Of the 907 respondents, 59% were male (Table 2). More than half of the respondents (57.1%) were from India, while the second-highest represented country was Pakistan, with 12.6%. Among respondents who answered the questions, 99.7% reported having been taught about antimicrobials, while 99.2% reported having been taught about AMR. Most were taught about AMR in broader microbiology (56%), pharmacology (43.8%), or medicine (24.7%) courses, with very few students (2.2%) reporting learning about it through internships.

**Table 2:** Demographic characteristics of the respondents.

Demographics	*n* (%)
Gender	
Male	535 (59.0)
Female	368 (40.6)
Prefer not to say	4 (0.4)
Country of origin	
Algeria	25 (2.8)
Egypt	50 (5.5)
India	518 (57.1)
Nigeria	94 (10.3)
Pakistan	115 (12.7)
Tunisia	16 (1.8)
Turkey	89 (9.8)

### 3.2 Knowledge and attitudes regarding AMU and AMR

#### 3.2.1 General scores on knowledge and attitudes

Overall, students had poor to average knowledge of AMS, AMU, and AMR, but held more positive attitudes overall (Table 3). The total mean percentage scores for knowledge and attitudes were 49.5 ± 17.0 and 65.3 ± 23.2, respectively. The lowest mean scores were observed for knowledge of AMS terms and policies, and the highest scores for positive attitudes toward factors influencing AMU.

**Table 3:** Mean and median percentage scores for knowledge and attitudes of participants.

Domain	Sub-categories	Percentage mean score ± SD	Percentage median score (IQR)
Knowledge	AMS	32.1 ± 22.7	36.4 (9.1–45.5)
	AMU	54.0 ± 22.2	55.0 (40.0–70.0)
	AMR	55.7 ± 22.6	62.5 (43.8–71.9)
	Total	49.5 ± 17.0	53.2 (40.4–61.7)
Attitudes	AMU	66.1 ± 22.9	74.9 (62.8–80.0)
	AMR	64.2 ± 25.2	74.3 (58.6–80.0)
	Total	65.3 ± 23.2	74.3 (61.9–80.2)

AMS, Antimicrobial Stewardship; AMU, Antimicrobial Use; AMR, Antimicrobial resistance; SD, Standard deviation.

#### 3.2.2 Knowledge regarding AMS

Students were tested on their knowledge regarding specific AMS terminologies and policies (Figure 1). There was a higher level of awareness (i.e., having heard of the term/policy before) than knowledge (i.e., being able to define the term/policy). Terms such as ‘antimicrobial resistance’, ‘therapeutic antibiotic use’, and ‘prophylactic antibiotic use’ were more familiar than ‘growth promoters’ and ‘HP-CIAs’. About a quarter (24.6%) were aware of ‘antimicrobial stewardship’, and only 20.8% could define the term. Students also exhibited generally poor awareness and knowledge of AMS-related policies. Only 30.5% stated that they were aware of antimicrobial restriction laws. Less than one-fifth (19.3%) were aware that there were national antimicrobial regulations guiding AMU in their countries, and only 6.6% could state at least one of these. Similarly, awareness and knowledge regarding international AMU guidance were also poor.

**Figure 1:**
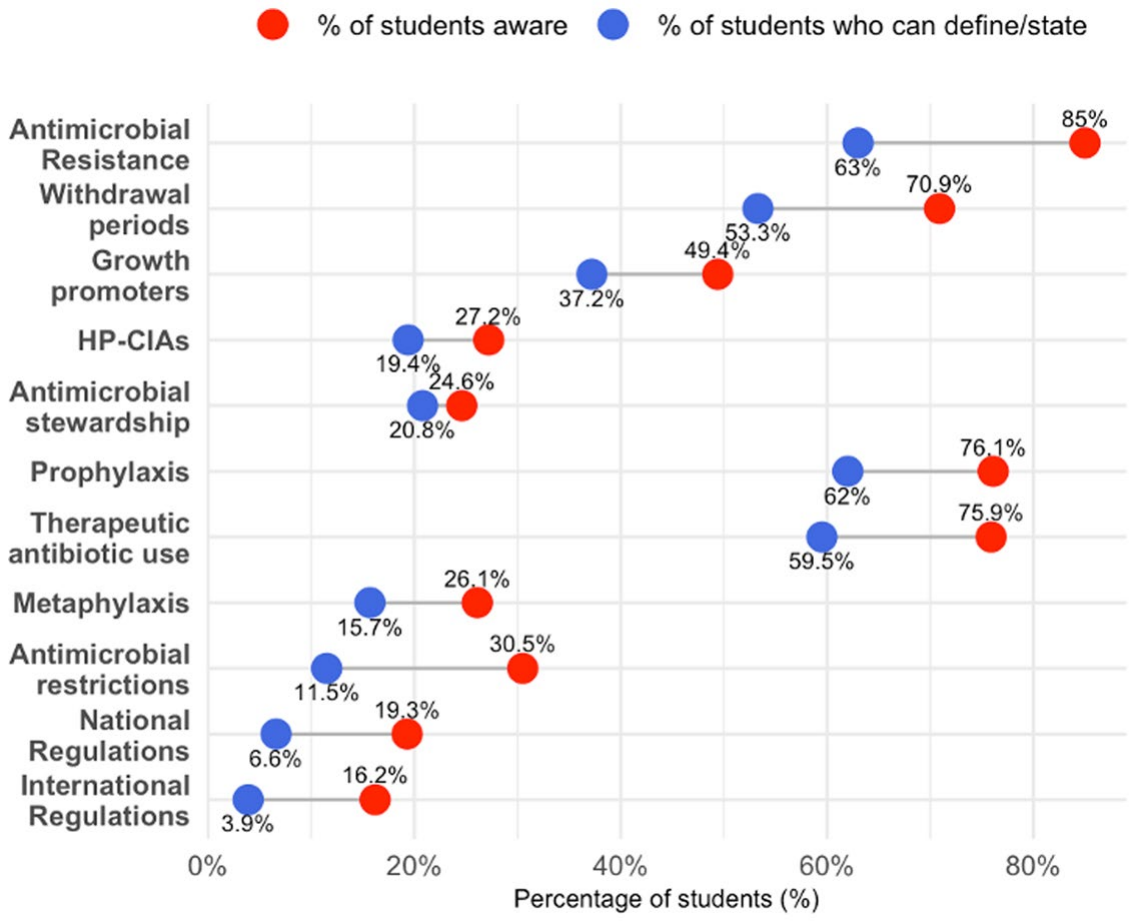
Awareness and knowledge regarding AMS terminology and policies.

Among students familiar with national AMU regulations, 50% stated they had learnt these from classroom lectures or textbooks, while 27.4% cited social media (e.g., newspapers) as their source of information (Table 4). Being able to mention national regulations was strongly associated with the students’ country of origin (*p*: 0.019), ranging from 1% (Turkey) to 10.4% (Pakistan). In addition, students who could mention regulations demonstrated significantly higher knowledge scores (OR: 4.74, 95% CI: 2.51–8.64, *p*: <0.001) than students who could not mention regulations.

**Table 4:** Knowledge sources regarding national and international antimicrobial guidance cited by participants.

Source of knowledge	National regulations, *n* (%) (*n* = 186)	International guidance, *n* (%) (*n* = 141)
Lectures/Textbooks	93 (50.0)	63 (44.7)
Internet/Media	51 (27.4)	48 (34.0)
Research journals	17 (9.1)	13 (9.2)
Government programmes/websites	9 (4.8)	2 (1.4)
Multinational organisations	7 (3.8)	15 (10.6)
Other	18 (9.7)	12 (8.5)
I do not know	6 (3.0%)	0 (0.0%)

#### 3.2.3 Knowledge on AMU and AMR

Most students (>80%) correctly stated that antibiotics could be used in treating bacterial infections, including salmonellosis, endometritis, and colibacillosis (Figure 2). However, fewer students were aware that antibiotics should not be used for viral diseases or infections, including chicken anaemia (44.1%), Marek’s disease (37.9%), and infectious bronchitis (25.1%). Similarly, only a few students knew that antibiotics should not be used for diseases of non-infectious or potentially non-infectious origin, including milk fever (53.5%), colic (40.6%), and lameness in large animals (38.6%).

**Figure 2:**
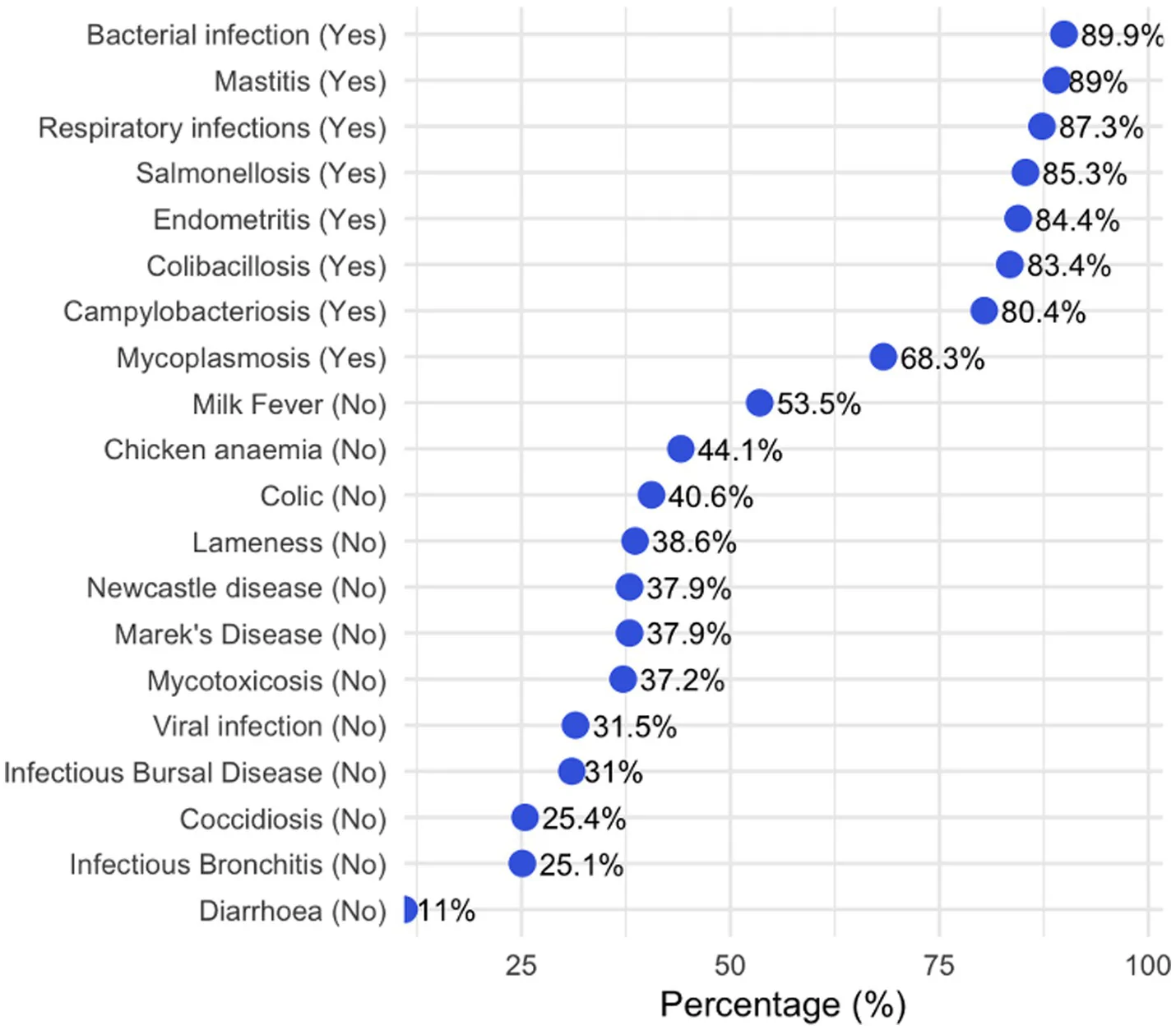
Students’ knowledge on antibiotic use for various conditions of veterinary concern.

Regarding AMR, 76.2% of students knew that AMU in livestock contributed to AMR in the veterinary field, while 70.2% understood that AMU in livestock could also contribute to AMR in humans (Table 5). Most (86.8%) correctly identified that bacteria could develop AMR, but fewer recognised that this was also true for viruses (36.0%), fungi (47.9%), and parasites (49.5%). Similarly, only 16.4% of students identified that animals could not develop AMR. However, 70% of students knew that veterinarians could play an important role in reducing AMR, while 76.3% understood that antimicrobial susceptibility tests (AST) were important for determining antibiotic effectiveness.

**Table 5:** Participants’ responses to AMU and AMR related questions.

Questions	Reference answer	Correct, *n* (%)	Incorrect, *n* (%)
What are antibiotics?	Definition of antibiotics	617 (68.0)	290 (32.0)
Does AMU in livestock contribute to AMR in the veterinary field?	Yes	691 (76.2)	216 (23.8)
Does AMU in livestock contribute to AMR in humans?	Yes	637 (70.2)	270 (29.8)
Is AMR a problem in the clinical treatment of humans?	Yes	706 (77.8)	201 (22.2)
Can bacteria develop AMR?	Yes	787 (86.8)	120 (13.2)
Can viruses develop AMR?	Yes	327 (36.0)	580 (64.0)
Can fungi develop AMR?	Yes	434 (47.9)	473 (52.1)
Can parasites develop AMR?	Yes	449 (49.5)	458 (50.5)
Can protozoa develop AMR?	Yes	440 (48.5)	467 (51.5)
Can animals develop AMR?	No	149 (16.4)	758 (83.6)
Can poultry develop AMR?	No	149 (16.4)	758 (83.6)
AST determines the likelihood of antibiotic effectiveness during bacterial infections.	True	692 (76.3)	215 (23.7)
Broad-spectrum antibiotics are recommended in the absence of an AST.	False	154 (17.0)	753 (83.0)
Veterinarians can play an important role in reducing AMR	True	635 (70.0)	272 (30.0)
It is not whether antibiotics should be used; it is the rational use that matters.	True	583 (64.3)	324 (35.7)
Higher antibiotic use in the veterinary field increases the risk of AMR.	True	634(69.9)	273 (30.1)

#### 3.2.4 Attitudes toward AMU

Students’ attitudes regarding AMU are presented in Figure 3. Overall, similar percentages of students (46.3 and 43.3%) agreed that antimicrobials are ‘most likely’ or ‘likely’ used for disease treatment and prevention. Similarly, about 42% of students considered that antimicrobials could be added to food and used for growth promotion.

**Figure 3:**
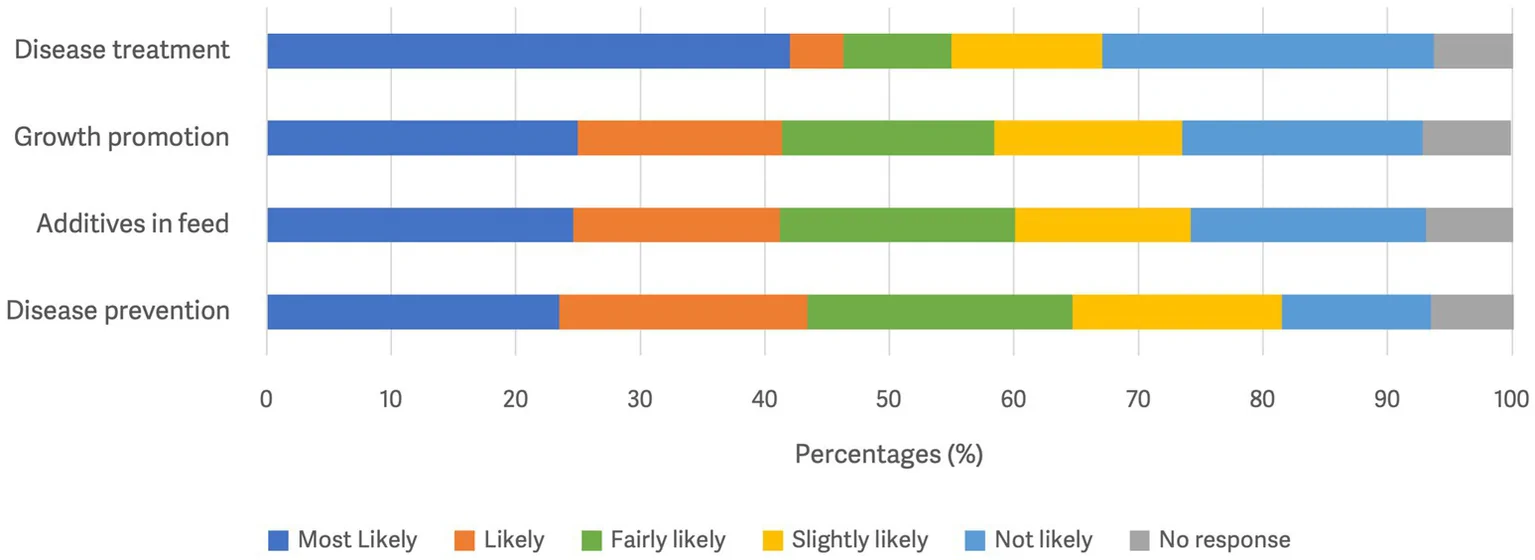
Participants’ attitudes regarding the use of antimicrobials.

Regarding factors influencing AMU, 75.2 and 71.6% of students considered disease severity and AST results to be important or very important, respectively (Figure 4). Only 32.1% of students considered that guidelines for prescribing antibiotics were very important for AMU. Factors ranked the least important included growth rate, owner’s desire, and product promotions. Students were asked about the importance of certain factors in influencing the decisions to conduct AST (Supplementary Figure 1). Overall, 49.4% of students agreed that animals not being responsive to previous medication was a very important factor. Repeated outbreaks, confirming clinical diagnosis, and disease conditions were also regarded as very important by 46.5, 43.0 and 42.1% of students, respectively. More than half (56.9%) stated that the cost of laboratory tests was an important or very important factor when deciding whether to conduct AST.

**Figure 4:**
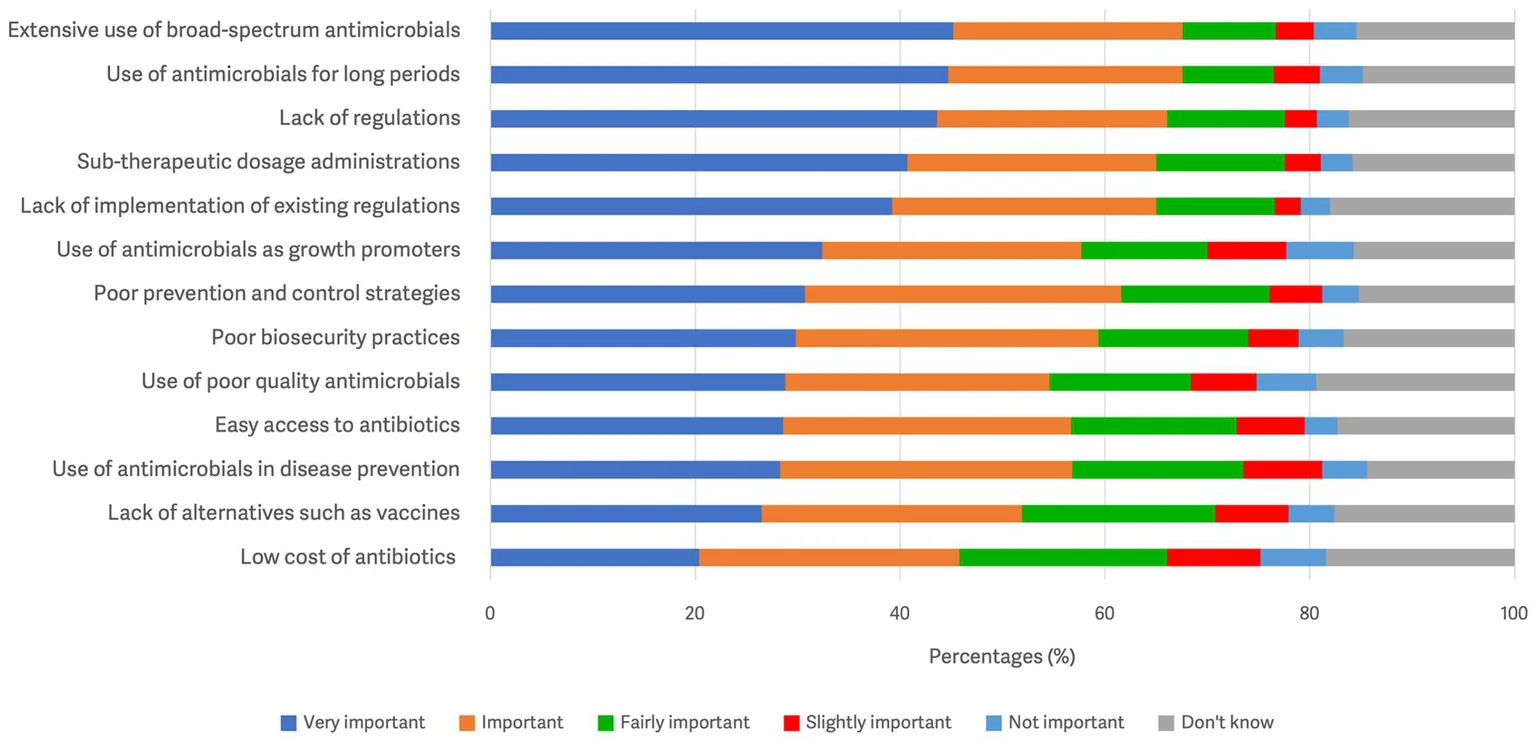
Participants’ attitudes regarding factors influencing AMU.

#### 3.2.5 Attitudes regarding AMR

The participants considered factors such as extensive use of broad-spectrum antimicrobials (67.5%), long-term use of antimicrobials (67.6%), and lack of regulations (66.3%) to be important or very important contributors to AMR (Figure 5). The factors regarded as the least important contributors included the use of antimicrobials in disease prevention (57%), lack of alternatives (51.9%), and low antibiotic cost (45.8%). To minimise AMR in veterinary medicine, increasing veterinary education and increasing public awareness were regarded as very important by 50.1 and 48.8% of respondents, respectively (Supplementary Figure 2). Conversely, the factors regarded as the least important included restricting antimicrobial prescribing (34.8%) and limiting the use of antibiotics to therapeutic use only (37.5%).

**Figure 5:**
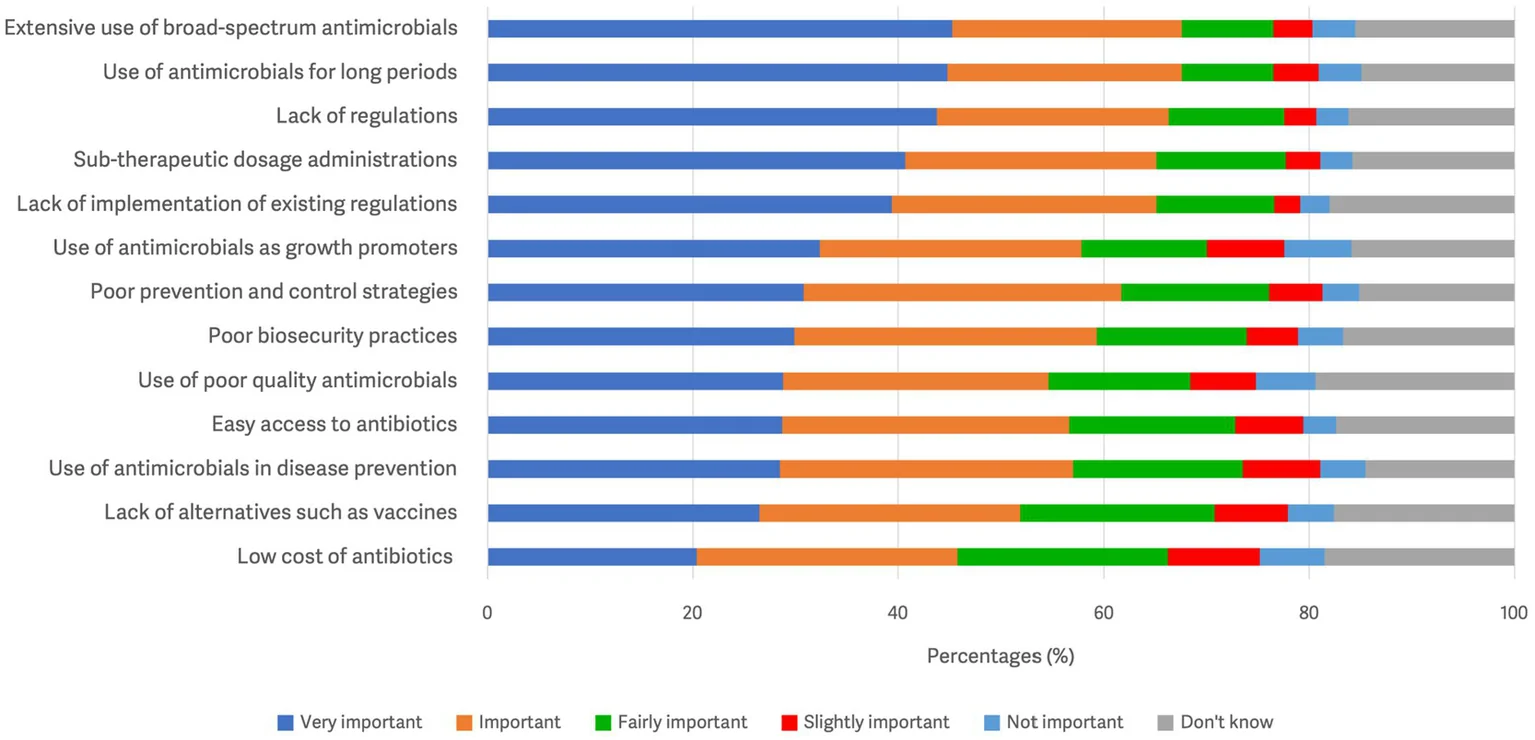
Attitudes regarding factors contributing to AMR.

### 3.3 Associations between demographics and awareness, knowledge, and attitudes

Bivariate and multivariate analyses were conducted to analyse associations between demographics and awareness, knowledge, and attitude scores (Tables 6, 7). Gender was not significantly associated with knowledge during bivariate analysis and thus was excluded from further inferential tests (Table 7). In the multivariate model, Nigerian students were significantly more likely to have better knowledge (aOR: 2.37, 95% CI: 1.09–5.55, p: 0.036) and attitude scores (aOR: 3.05, 95% CI: 1.11–8.62, p: 0.031) than Egyptian students. Conversely, Indian (aOR: 0.14, 95% CI: 0.06–0.34, p: <0.001) and Turkish students (aOR: 0.09, 95% CI: 0.01–0.37, p: 0.003) were less likely to have good knowledge than Egyptian students. Turkish students were also less likely to have positive attitudes than Egyptian students (aOR: 0.25, 95% CI: 0.11–0.55, *p* < 0.001).

**Table 6:** Bivariate analysis (Pearson’s chi-square test) to determine independent associations between scores and demographics.

Category	Knowledge				Attitude			
	Satisfactory *n* (%)	Unsatisfactory *n* (%)	*χ* ^2^	*p*-value	Satisfactory *n* (%)	Unsatisfactory *n* (%)	*χ* ^2^	*p*-value
Gender								
Male	47 (8.8)	488 (91.2)	0.13 (1)	0.720	312 (58.3)	223 (41.7)	2.95 (1)	**0.091**
Female	29 (7.9)	339 (92.1)			236 (64.1)	132 (35.9)		
Country								
Algeria	1 (4.0)	24 (96.0)	129.1 (6)	**<0.001**	16 (64.0)	9 (36.0)	61.2 (6)	**<0.001**
Egypt	10 (20.0)	40 (80.0)			40 (80.0)	10 (20.0)		
India	18 (3.5)	500 (96.5)			283 (54.6)	235 (45.4)		
Nigeria	35 (37.2)	59 (62.8)			86 (91.5)	8 (8.5)		
Pakistan	11 (9.6)	104 (90.4)			75 (65.2)	40 (34.8)		
Turkey	2 (2.2)	87 (97.8)			42 (47.2)	47 (52.8)		
Tunisia	1 (6.2)	15 (93.8)			9 (56.3)	7 (43.8)		

Bolded numbers indicate statistical significance.

**Table 7:** Multivariate analysis (logistic regression) determining the association between demographics and scores.

Category	Good knowledge		Positive attitudes	
	aOR (95% CI)	*p*-value	aOR (95% CI)	*p*-value
Gender				
Male	r			
Female	N/A*		**1.360 (1.017–1.822)**	**0.038**
Country				
Egypt	r			
Algeria	0.167 (0.001–0.952)	0.097	0.478 (0.161–1.411)	0.178
India	**0.144 (0.063–0.343)**	**<0.001**	**0.327 (0.151–0.647)**	**0.002**
Nigeria	**2.372 (1.085–5.551)**	**0.036**	**3.048 (1.111–8.621)**	**0.031**
Pakistan	0.423 (0.166–1.088)	0.070	0.531 (0.227–1.161)	0.125
Turkey	**0.092 (0.014–0.368)**	**0.003**	**0.251 (0.106–0.550)**	**<0.001**
Tunisia	0.267 (0.014–1.578)	0.226	0.323 (0.095–1.097)	0.067

*Excluded from the logistic regression due to bivariate analysis results. ‘r’ represents the reference category for the analysis. Bolded numbers represent statistical significance. aOR, Adjusted odds ratio.

## 4 Discussion

Future veterinarians play an important role in governing AMU in animals, and their knowledge and attitudes regarding AMU, AMR, and AMS can critically influence their eventual future practice behaviours (5). To our knowledge, this is the first study exploring veterinary students’ knowledge and attitudes toward AMU, AMR, and AMS across multiple countries. Some countries represented in this study are known for high levels of AMU in the veterinary sector. For instance, Nigeria is also projected to have one of the greatest percentage increases in AMU globally by 2030 (27). In addition, global antibiotic consumption increased by 65% between 2000 and 2015, and Tunisia and Turkey were classified among countries with the highest consumption rates (28). These patterns underscore the importance of understanding the knowledge and attitudes of veterinary students from these countries. The inclusion of students across seven countries and 43 higher VEEs allows for the identification of the most prevalent knowledge across diverse settings. The survey was deployed in 2021 across most participating countries; however, due to administrative and institutional logistics within NetVet4SAMU, data collection in Tunisia and Algeria was completed in 2024. This temporal gap introduces a variation in the educational context of the respondents. The 2021 cohorts completed their training at the height of the COVID-19 pandemic, a period documented for severe disruptions in practical veterinary competencies and clinical AMU exposure (29). On the other hand, the 2024 cohorts graduated during a stabilized, post-pandemic educational environment. As the majority of our participants experienced COVID-induced study disruptions, students’ AMU knowledge may reflect study disruption they may have faced during the COVID era. More studies are indicated to establish definitive associations between the pandemic and students’ practical knowledge.

It is concerning that only 2.2% of students who learned about AMR did so from internships. Veterinary internships and extramural placements are a core requirement of most veterinary curricula, allowing students to experience real-life cases in professional environments while enhancing their practical training (30, 31). Thus, it presents a valuable opportunity to understand the risks of AMU and AMR. Only a minority of students learned about AMR during these placements, suggesting either a lack of placements in veterinary school or a potential lack of emphasis on AMS during clinical rotations.

Most students were familiar with the term ‘AMR’ and could define it accurately. However, there was generally low knowledge regarding other terms, including AMS, HP-CIAs, and metaphylactic AMU. Similarly, only 17% of final-year veterinary students in a Nigerian study reported being familiar with the term ‘AMS’ (32). This may indicate that students are not being presented with a thorough understanding of AMS during lectures. The majority of students were also unfamiliar with national and international AMU policies. Our findings contrast with studies in Australia and the United States, where veterinary students were aware of at least one AMS guideline, albeit most remained generally unaware of the full range of guidelines in existence (33, 34) In our study, there was a significant association between students’ being able to mention national regulations and their country of origin. It must be noted, though, that across all countries, less than 20% of students were familiar with the guidelines, even though most students reported learning about them during their education. Thus, guidelines may be inconsistently taught both within and across countries. It is also likely that guidelines were not as emphasised as those for other topics, since AMR was primarily taught as a subtopic to other core veterinary subjects rather than as a standalone module. According to past studies, veterinary students often focus their studies on topics they are more likely to be examined on due to their high workload, leading to uneven retention of the syllabus across students (35, 36). A significant percentage of students also learned of these regulations through research journals and the internet. Thus, VEEs that encourage students to rely on external knowledge sources may be associated with higher rates of AMR awareness.

Regarding appropriate AMU, a significant percentage of students considered it acceptable to use for growth promotion, disease prevention and to add them to feed. The use of antibiotics as growth promoters is popular across Asian countries, including China and India (11, 37). These practices have also been documented in countries such as Egypt and Nigeria (38, 39). As these students contribute the majority here, their exposure to these practices might influence their beliefs about using antibiotics in this manner. However, recent evidence indicates that antibiotics do not contribute significantly to feed conversion efficiency but rather cause resistance selection (37). Only 20–50% of students were aware that antibiotics were inappropriate for viral and non-infectious diseases. This reflects similar findings in previous studies, where up to one-third of veterinary students in a Bangladeshi study incorrectly stated that antibiotics are directly effective against viruses, and 23% of veterinarians in a Bhutan study believed that antibiotics were effective for all infectious diseases (40, 41). The perception of antibiotics as a cure-all is widespread, particularly among people without a medical background (42). If veterinary medical students share these views, it may influence their future practices, bearing grave implications for animal and human health. Interestingly, most students were able to correctly define antibiotics as a drug effective only against bacterial infections. This indicates that some students may understand that antibiotics are only effective for bacterial diseases, but may be unsure as to the infectious aetiology of some common veterinary diseases. Thus, it is important for curricula to differentiate between bacterial and non-bacterial diseases and emphasise that antibiotics are effective only for bacterial diseases. In addition, the guidelines set out by WOAH’s Terrestrial Animal code, such as confirmatory diagnosis prior to AMU and ensuring dosage completion, should also be introduced in classrooms (15).

Students demonstrated better knowledge of AMR, including its clinical implications across human and animal sectors and the importance of veterinarians in reducing AMR risks. However, fewer students were aware that other pathogens, including viruses, fungi, and parasites, could develop AMR. Similarly, a Norwegian study found that final-year veterinary students demonstrated lower knowledge of managing clinical cases of fungal AMR compared with bacterial AMR (43). Currently, there is a higher emphasis on bacterial resistance over other pathogens with respect to AMR, as antibacterials are the most commonly used class of antimicrobials (44). This might have contributed to students’ low awareness of resistance in other pathogens. Only 17% knew it was not the animals themselvesw that developed AMR, but rather the pathogens that may infect them, with findings similar to a United Kingdom study in which a significant proportion of veterinary students believed that it was the animals that developed resistance to antibiotics (45). Students’ responses here indicate their awareness of the basic concept of AMR but insufficient information regarding its broader impact.

Attitudes toward AMU were positive, with students indicating that they considered disease severity and results of AST as important in influencing AMU. This demonstrates students’ awareness of the importance of laboratory diagnosis in ensuring AMS. More than half of students also considered the cost of laboratory analysis as an influencing factor in conducting AST. This raises important considerations of cost and access, particularly in communities and countries where laboratory analysis may be limited and AST may not be carried out or interpreted using appropriate breakpoints. Practising veterinarians from these countries may not be able to conduct diagnostic testing or AST even while being aware of its importance in ensuring AMS (46). Guidelines influencing prescribing were considered less important factors in AMU and AMS, likely because most students are unaware of these regulations. This aligns with a recent study in the United States, which indicated poor adherence among veterinarians to institutional guidelines in a teaching hospital (47). In contrast, WOAH emphasises that veterinarians’ responsibilities should include minimising AMU whenever necessary by adhering to guidelines, such as promoting good animal husbandry, prudently selecting antimicrobials, and observing withdrawal periods (15). It is therefore necessary to educate students regarding the importance of consulting guidelines prior to AMU in their future practices.

Students’ perspectives regarding AMR and AST further highlight areas that require curricular reform. While about 45% of students were aware that broad-spectrum antimicrobials and AMU for long periods were very important contributors to AMR, only 28% considered that using antimicrobials for disease prevention was a very important factor. In addition, the factors ranked lowest by students for minimising AMR included improving animal husbandry (68%) and limiting antibiotic prescription for therapeutic use (63%). Similarly, only 56% of veterinary students in a study in South Africa agreed or strongly agreed that banning prophylactic prescription would reduce AMR (48). The misuse of antimicrobials for prophylaxis is considered by WOAH one of the most important contributing factors to AMR in veterinary medicine; thus, it is important that students are aware of the dangers of such use. Students may also be informed about the necessity of alternatives to antimicrobials and preventive management in reducing disease outbreaks and subsequently AMU (15). The step considered most important in minimising AMR was improving veterinary education in this area. Similarly, studies in Bangladesh and Nigeria also report high positive attitude scores for AMU and AMR (5, 40). This reflects an encouraging enthusiasm among students to learn about AMS.

Multivariate analysis found no associations with gender, but more significant associations by country of origin. While this might be due to differences in national curricula, this study included students from 43 universities across 7 countries, and these VEEs may also differ in their curricular emphasis on AMS, even within the same country. In addition, other factors, including the timing of data collection, students who chose to participate in this study, and language barriers, may have also contributed to the differing levels of knowledge and attitudes across students. Future studies may evaluate curricula within and across countries to identify replicable models for AMR and AMU inclusion. For instance, Nigerian and Egyptian students exhibited higher knowledge and more positive attitudes toward AMS. This is comparable to a KAP study conducted across three African countries, in which Nigerian veterinary students were observed to have better perceptions of AMS than Sudanese students (49). Paradoxically, Nigeria and Egypt also report high levels of antimicrobial misuse and resistance in the animal sector, raising questions of why this knowledge does not translate into practice. A possible reason for this may include the enforcement of regulations guiding AMU in field practice, particularly among non-licensed professionals (38, 50). Still, evaluating curricula from countries where students exhibit higher levels of knowledge can provide useful information for a harmonised global AMR curriculum for veterinary and human medical students.

### 4.1 Study limitations

Most study respondents were from India and Pakistan, reflecting the higher number of responses received from these countries during questionnaire deployment. This disparity may reduce the precision of adjusted estimates, particularly for countries with fewer participants. Due to resource constraints, the questionnaire was administered online, thereby raising the potential for digital access bias. Data was collected over a three-year period and data from Algeria and Tunisia were collected latter than from other countries. This counts as a study limitation, as the variation may have affected students’ exposure to stewardship education particularly in the light of the COVID pandemic. In addition, the variation in sample sizes across countries, and potential clustering of participants within VEEs, may limit the generalisability and statistical precision of the study. As most respondents had access to the internet, they may have consulted external sources while answering the questions, hence, their scores may not necessarily reflect their actual knowledge. However, our findings provide important insights for AMR inclusion in veterinary curricula.

## 5 Conclusion

Our study examined the knowledge and attitudes of veterinary students regarding AMU, AMR, and AMS across 7 countries and 43 VEEs. The study revealed notable gaps in students’ understanding of AMS concepts and guidelines, appropriate AMU, and the resistance potential of non-bacterial pathogens. However, most students demonstrated an acceptable understanding of AMR and the importance of veterinarians in curtailing this problem. Based on these findings, we recommend the deliberate integration of AMR and AMS into national veterinary curricula, either as a dedicated module or through a longitudinal approach that reinforces these concepts throughout students’ veterinary education. This should be done within existing WOAH Day-1 competencies relating to the appropriate use of antimicrobials in infectious disease control. The curricula should also emphasize national and international AMS guidelines, proper AMU, and the importance of preventive practices. Experiential learning opportunities, including internships and external clinical placements, should also be leveraged to strengthen students’ understanding of AMS in real application settings. Case and contextual studies can also be used in teaching AMU during classes to increase authenticity during learnings. Further research can focus on evaluating veterinary curricula across countries, highlighting effective inclusions of AMR, and understanding the influence of external sources in improving AMR awareness. National governmental bodies can also strengthen AMU regulations and policies and increase awareness of AMR among veterinary professionals and animal health workers to improve current field practices.

## Data Availability

The datasets presented in this article are not readily available because the data that support the findings of this study will be available from the corresponding author only upon reasonable request. Requests to access the datasets should be directed to mahmoud.eltholth@rhul.ac.uk.
