# Supplementary material for: Knowledge and attitudes toward antimicrobial use, resistance, and stewardship among final-year veterinary students: a multinational cross-sectional survey
Source: Front Vet Sci. 2026 Oct 6;13:1957292. doi: 10.3389/fvets.2026.1957292 (PMC13681828; doi:10.3389/fvets.2026.1957292)
Supplement: Supplementary file 1 [file Data_sheet_1.pdf]

Final Year Veterinary Students_KAP Survey_Actual

Page 1: Knowledge and perception of antimicrobial use and antimicrobial resistance among final year veterinary students

Invitation to Participate:

Antimicrobial resistance (AMR) is an urgent global one health challenge and irrational and over use of antimicrobials in livestock production and human medicine contributing to this problem.

This questionnaire is part of a large interdisciplinary research project on the drivers for AMR and the role of livestock and poultry production in India (see www.liverpool.ac.uk/infection-and-global-health/research/darpi/) involving multiple UK and Indian partners, with this specific survey led by the University of Liverpool, University of Edinburgh and the Karnataka Veterinary, Animal and Fisheries Sciences University. This study is designed to help us understand how and what veterinary students are taught, their knowledge and attitudes around AMR and antimicrobial use.

You have been invited to participate in this study because you have recently graduated or are near to the completion of your veterinary course and will be a future prescriber and therefore represent a important stakeholder.

What questions are we asking?

We will be asking questions about antimcirobial use (AMU) and AMR in the context of your veterinary education and experience. We will also be asking a wide range of questions concerning your knowledge and attitudes towards AMU and AMR.

Will my participation be kept confidential?

Participation is entirely voluntary and you do not have to take part in the study, or give any reasons if you do not wish to take part, but you must be at least 18 years old to

participate. All data will be confidential and we will be asking for information about where you live, age, gender, and the university where you undertook your veterinary education, however these will be assigned a numerical code by researchers to subsequently anonymise this information in all our data analysis.

What will happen to the results of the study?

We will provide a summary of the findings to the participating univeristies, as well as through social media. The results will also be important for directing future research on the role of veterinary education in good antimicrobial stewardship and in preventing AMR.

Depending upon the results, we may also publish them in a suitable veterinary journal.

The survey should take no longer than 25 minutes.

What next?

If you wish to take part in the survey then please tick the box below to indicate your informed consent. You are free to withdraw from the questionnaire at any time until you have selected the finish option on the final page of the questionnaire. After this time it will not be possible to withdraw your responses.

Please answer the questions by selecting the most appropriate answer box(es), or typing your answer in as much detail as possible.

What if I am unhappy or there is a problem?

If there are any problems or you have any issues regarding this study, please let us know by contacting Professor Williams (njwillms@liverpool.ac.uk), and we will try to help. If you remain unhappy or have a complaint which you feel cannot come to us with then you should contact the Research Ethics and Integrity Office at the University of Liverpool and citing the name of this survey and email your complaint to ethics@liv.ac.uk.

1. **Do you agree to take part of this research? By ticking yes below, you are providing informed consent to participate in the survey.** ✱ *Required*

- ○ Yes
- ○ No

1.a. If No, could you please explain why?

Page 2: 1. Background information (Please complete the following information about yourself)

2. Country

3. University/Faculty/College name

4. What is your Date of Birth?

Dates need to be in the format 'DD/MM/YYYY', for example 27/03/1980.

(dd/mm/yyyy)

5. What is your gender?

- Male
- Female
- Other

6. What is the date of your graduation?

Dates need to be in the format 'DD/MM/YYYY', for example 27/03/1980.

(dd/mm/yyyy)

7. What is the name of the course/degree you are undertaking or have recently completed?

8. Please state the number of years you have been in the course

Times need to be in the format 'HH:MM', for example 15:43.

(hh:mm)

9. What area of veterinary medicine are you interested in for your future career, *(Please select all that apply)*?

- ☐ Equine
- ☐ Ruminants
- ☐ Poultry
- ☐ Companion animals
- ☐ Public Health
- ☐ Education/Research
- ☐ Haven't decided yet
- ☐ Other

9.a. If you selected Other, please specify:

Page 3: 2. Knowledge and perception of antimicrobials and antimicrobial use

10. In which year and subject/s were you taught about antimicrobials?

	Subject/s name in which you have been taught about antimicrobials
1st Year	
2nd Year	
3rd Year	
4th Year	
5th Year	

11. What do you understand by the word **antimicrobial**?

12. What do you understand by the word **antibiotic**?

13. What do you think is the difference between the terms **antibiotic** and **antimicrobial**?

14. In your opinion, which characteristics of antibiotics are important for determining **dosing regimes**?

15. In your opinion, which characteristics of antibiotics are important for determining the **route of administration**?

16. Have you heard of “**Highest Priority Critically Important Antimicrobials (HP-CIA)**” list by the World Health Organisation (WHO)?

- Yes
- No

16.a. If yes, which antimicrobials are **HP-CIAs** according to the WHO classification?

17. Have you heard about "**Prophylactic**" use of antimicrobials?

- Yes
- No

17.a. If yes, what does "**Prophylactic**" use of antimicrobials mean to you?

17.b. Can you please give examples of when you think **prophylactic use** of antimicrobials in animals is appropriate ?

18. Have you heard about "**Metaphylactic**" use of antimicrobials?

- Yes
- No

18.a. If yes, what does "**Metaphylactic**" use of antimicrobials mean to you?

18.b. Can you please give examples of when you think **metaphylactic use** of antimicrobials in animals is appropriate?

19. Have you heard about "**Therapeutic**" use of antimicrobials?

- Yes
- No

19.a. If yes, what does "**Therapeutic**" use of antimicrobials mean to you?

19.b. Can you please give examples of when you think **therapeutic use** of antimicrobials in animals is appropriate?

20. In your opinion, what are antimicrobials used for? (*Please rate from 1 to 5 in order of how antibiotics are used in Livestock – 1 being most likely to be used and 5 least likely) (1= Most likely; 2=Slightly likely; 3=Fairly likely; 4=likely; 5 = least likely)*

	1= Most likely	2=Slightly likely	3=Fairly likely	4=likely	5 = least likely
Treatment of diseased animals/birds	○	○	○	○	○
Growth promotion	○	○	○	○	○
Preventing diseases	○	○	○	○	○
Feed additives	○	○	○	○	○

21. For which of the following health conditions do you think antimicrobials can be used in **livestock**? *If you are not very confident then please select "I don't know".*

	Yes	No	I don't know
Mastitis	○	○	○
Milk fever	○	○	○
Lameness	○	○	○
Colic	○	○	○
Diarrhoea	○	○	○
Respiratory tract infection	○	○	○
Endometritis	○	○	○
Viral infection	○	○	○
Bacterial infection	○	○	○
Other conditions	○	○	○

22. For which of the following health conditions do you think antimicrobials can be used in **poultry**? *If you are not very confident then please select "I don't know".*

	Yes	No	I don't know
Colibacillosis	○	○	○

Salmonellosis	○	○	○
Campylobacteriosis	○	○	○
Mycoplasmosis	○	○	○
Mycotoxicosis	○	○	○
Newcastle disease	○	○	○
Marek's disease	○	○	○
Infectious bronchitis	○	○	○
Infectious bursal disease	○	○	○
Chicken anaemia	○	○	○
Coccidiosis	○	○	○
Other conditions	○	○	○

23. In your opinion, what are the **factors influencing prescribing/using** of a specific type of antimicrobial? *(Please rate the importance of each of the following factors on a scale of 1 – 5 (1= Not important; 2=Slightly important; 3=Fairly important; 4=Important; 5 =Very important), if you are not very confident then please select "I don't know".*

	1= Not important	2=Slightly important	3=Fairly important	4=Important	5 =Very important	6=I don't know
Disease condition/severity (clinical symptoms)	○	○	○	○	○	○
Perceived or expected outcome to the herd/flock	○	○	○	○	○	○
Results of laboratory diagnosis	○	○	○	○	○	○
Cost of the antibiotic	○	○	○	○	○	○
Owners' desire	○	○	○	○	○	○

Availability of the antibiotic						
Route of administration						
Results of antibiotic sensitivity test						
Ease of administration						
Withdrawal period						
Economic status of the owner						
Veterinary education/training						
Product promotions						
Guidelines/policies for prescriptions						
Growth rate/feed conversion ratio						
Failure of previous medication						
Cost-effectiveness (is it cost effective to treat the animal/poultry with this specific antibiotic or not)						
Animal species						
Site of infection						

24. What are the factors influencing the decision of submitting samples for

culture/antibiotic sensitivity test before prescribing antimicrobials? *(Please rate the importance of each of the following factors on a scale of 1 – 5 (1= Not important; 2=Slightly important; 3=Fairly important; 4=Important; 5 =Very important) . If you are not very confident then please select "I don't know".*

	1= Not important	2=Slightly important	3=Fairly important	4=Important	5 =Very important	6=I don't know
Disease Condition/Severity (clinical symptoms)	◯	◯	◯	◯	◯	◯
Perceived or expected outcome to the herd/flock	◯	◯	◯	◯	◯	◯
Availability of diagnostic labs	◯	◯	◯	◯	◯	◯
Costs of laboratory analysis	◯	◯	◯	◯	◯	◯
Cost of the antibiotic	◯	◯	◯	◯	◯	◯
Owners' desire	◯	◯	◯	◯	◯	◯
Ease of taking samples	◯	◯	◯	◯	◯	◯
Repeated infections/outbreaks	◯	◯	◯	◯	◯	◯
Economic status of the animal/poultry owner	◯	◯	◯	◯	◯	◯
Veterinary educations/training	◯	◯	◯	◯	◯	◯
Guidelines/policies for prescriptions	◯	◯	◯	◯	◯	◯
To confirm clinical diagnosis	◯	◯	◯	◯	◯	◯

The scale of the infection/outbreak	○	○	○	○	○	○
Species/breed/line of the animal/bird	○	○	○	○	○	○
Not responsive to previous medication	○	○	○	○	○	○

25. Are you aware of any national regulations for the use of antimicrobials in the veterinary field?

- ○ Yes
- ○ No

25.a. If Yes, please state?

[ ]

25.b. How did you find out about these regulations?

[ ]

26. Are you aware of any international regulations for the use of antimicrobials in the veterinary field?

- ○ Yes
- ○ No

26.a. If Yes, which ones?

26.b. How did you find out about these regulations?

27. Are you aware of any restrictions for use of certain antimicrobials for livestock/poultry?

- Yes
- No

27.a. If Yes, what are these restrictions and what are the list of restricted products?

28. Have you ever heard of the term '**antibiotic/antimicrobial stewardship**'?

- Yes
- No

28.a. What do you think '**antibiotic/antimicrobial stewardship**' means?

29. Have you heard about “Growth Promotion”?

- Yes
- No

29.a. If yes, what do you think is the definition of “Growth Promotion”?

29.b. What do you think are the mechanisms of action of “Growth Promoters”/how do “Growth Promoters” work?

29.c. In your opinion what products can be used as “Growth Promoters”?

29.d. In your opinion, what are the advantages of “Growth Promoters”?

29.e. In your opinion, what are the disadvantages of “Growth Promoters”?

30. Have you heard about “Withdrawal Period for antimicrobials”?

- ◯ Yes
- ◯ No

30.a. If yes, what do you think is the definition/meaning of “Withdrawal Period for antimicrobials”?

31. In the veterinary field, who do you think should be allowed to prescribe antibiotics?

32. Other than veterinarians, do you know whether other people prescribe/recommend antimicrobials to be given to livestock/poultry? (i.e farmers, paravets, non-vet farm supervisors etc).

33. Where do you think that these people learn or gain knowledge on which antibiotics to prescribe/recommend?

Page 4: 3. Knowledge and perception towards antimicrobial resistance (AMR)

34. In your studies, have you heard of the term **“Antimicrobial Resistance (AMR)/Antibiotic Resistance (ABR)”**?

- Yes
- No

35. In which year and subject/s were you taught about “Antimicrobial Resistance (AMR)/Antibiotic Resistance (ABR)”?

	Subject/s name in which you have been taught about antimicrobials
1st Year	
2nd Year	
3rd Year	
4th Year	
5th Year	

36. In your opinion, what is **AMR/ABR**?

37. Do you think that the use of antimicrobials for livestock/poultry contributes to the emergence of antimicrobial resistance in livestock/poultry?

- Yes
- No
- I don't know

38. Do you think that the use of antimicrobials for livestock/poultry contributes to the emergence of antimicrobial resistance in humans?

- Yes
- No
- I don't know

39. What do you think are the factors contributing to the emergence/development of AMR in veterinary medicine? *(Please rate the importance of each of the following factors on a scale of 1 – 5 (1= Not important; 2=Slightly important; 3=Fairly important; 4=Important; 5 =Very important) if you are not very confident please select "I don't know".*
.

	1= Not important	2=Slightly important	3=Fairly important	4=Important	5 =Very important	6=I don't know
Use of antimicrobials as growth promoters in livestock/poultry food production	○	○	○	○	○	○
Use of antimicrobials to prevent diseases in healthy livestock/poultry	○	○	○	○	○	○

Poor prevention and control strategies for livestock/poultry diseases	◌	◌	◌	◌	◌	◌
Sub-optimal/sub-therapeutic dosage of antimicrobials in livestock/poultry	◌	◌	◌	◌	◌	◌
Using antimicrobials for long periods in livestock/poultry	◌	◌	◌	◌	◌	◌
Use of poor quality antimicrobials in the veterinary field	◌	◌	◌	◌	◌	◌
Lack of regulations for use of antimicrobials in the veterinary field	◌	◌	◌	◌	◌	◌
Lack of implementation of existing regulations for the use of antimicrobials in the veterinary field	◌	◌	◌	◌	◌	◌
Poor biosecurity practices (Poor farm/animal/poultry shed hygiene)	◌	◌	◌	◌	◌	◌
Extensive use of broad-spectrum antimicrobials for livestock/poultry	◌	◌	◌	◌	◌	◌

Low cost of antibiotics in comparison to other treatment regimens for livestock/poultry	○	○	○	○	○	○
Easy accessibility of antibiotics for livestock/poultry	○	○	○	○	○	○
Lack of alternatives such as vaccines for livestock/poultry diseases.	○	○	○	○	○	○

40. Do you think AMR is a problem in terms of clinical treatment in livestock/poultry?

- ○ Yes
- ○ No
- ○ I don't know

41. Which of the following do you think can develop resistance against antimicrobials?

	Yes	No	I don't know
Bacteria	○	○	○
Viruses	○	○	○
Fungi	○	○	○
Parasites	○	○	○
Protozoa	○	○	○
Animals	○	○	○

Poultry	○	○	○

42. In your opinion, how do pathogens develop resistance against antimicrobials (what are the mechanisms of developing antimicrobial resistance)?

43. In your opinion, what steps can be taken to minimise/prevent the development/emergence of AMR? *(Please rate the importance of each of the following factors on a scale of 1 – 5 (1= Not important; 2=Slightly important; 3=Fairly important; 4=Important; 5 =Very important) if you are not very confident please select "I don't know".*

	1= Not important	2=Slightly important	3=Fairly important	4=Important	5 =Very important	6=I don't know
Veterinary education should stress more on AMU and AMR	○	○	○	○	○	○
Increasing veterinarians' awareness of the problem	○	○	○	○	○	○
Increasing farmers' awareness of the problem	○	○	○	○	○	○
Restricting prescriptions of antimicrobials for livestock/poultry	○	○	○	○	○	○

Improving animal husbandry and hygiene	○	○	○	○	○	○
Obligatory vaccination programmes for livestock and poultry	○	○	○	○	○	○
Improving regulations for productions and trading of veterinary health products	○	○	○	○	○	○
Improving diagnosis of livestock and poultry diseases	○	○	○	○	○	○
Increasing public awareness around AMU in livestock/poultry and the risk of AMR	○	○	○	○	○	○
Encouraging veterinary professionals to conduct susceptibility/sensitivity testing before prescribing antibiotics	○	○	○	○	○	○
Only dispensing the required quantity of the antibiotics for the full course of treatment of livestock/poultry	○	○	○	○	○	○
Limiting the use of antibiotics in the veterinary field to the therapeutic use only	○	○	○	○	○	○

44. Is there anything else you think that is important in preventing the emergence of

AMR?

45. For the following statements, can you please either choose true or false? *If you are not very confident please select “I don’t know”.*

	True	False	I don't know
The ability of an Antibiotic agent to control causative agents is dependent upon its duration of action and the concentration that the drug attains at the infection site.	○	○	○
Antibiotic susceptibility test helps to determine the likelihood that a particular antibiotic will be effective in treatment of a certain bacterial infection.	○	○	○
For the treatment of bacterial infections, broad spectrum antibiotics are highly recommended in the absence of a susceptibility test.	○	○	○
The more antibiotics are used in the veterinary field, the higher is the risk that resistance develops and spreads	○	○	○
Veterinarians can play an important role in reducing the emergence and spread of AMR	○	○	○
It is not whether antibiotics should be used or not, but the rational use of antibiotics that matters	○	○	○

Page 5: Final page

Thank you for completing the survey
