# Supplementary material for: Knowledge and attitudes toward antimicrobial use, resistance, and stewardship among final-year veterinary students: a multinational cross-sectional survey
Source: Front Vet Sci. 2026 Oct 6;13:1957292. doi: 10.3389/fvets.2026.1957292 (PMC13681828; doi:10.3389/fvets.2026.1957292)
Supplement: Supplementary file 2 [file Table_1.docx]

**Supplementary Table 1:** Countries and VEEs included in the survey.

| Country | National population size | No of accredited VEEs | No of selected VEEs. | No. and names of selected |
| --- | --- | --- | --- | --- |
| Algeria | ≈ 48 million (2023 population estimate) | 6 | 1 | Institute of Veterinary Sciences, University Frères Mentouri Constantine. |
| Egypt | ≈ 120 million (2017 census) | 17 | 5 | Faculty of Veterinary Medicine, Cairo University. |
|  |  |  |  | Faculty of Veterinary Medicine, Mansoura University |
|  |  |  |  | Faculty of Veterinary Medicine, Benha |
|  |  |  |  | Faculty of Veterinary Medicine, Aswan University |
|  |  |  |  | Faculty of Veterinary Medicine, Menoufia University |
| India | ≈1.5 billion (census 2023) | 59 | 15 | College of Veterinary Sciences, Lakhimpur, Assam Agricultural University |
|  |  |  |  | Bangalore Veterinary College, Karnataka Veterinary Animal and Fisheries Sciences University |
|  |  |  |  | College of Veterinary Sciences, Khanapara, Assam Agricultural University |
|  |  |  |  | NTR College of Veterinary Sciences, Gannavaram, SV Veterinary University |
|  |  |  |  | Veterinary College, Nagpur, Maharashtra Animal & Fisheries Sciences University |
|  |  |  |  | College of Veterinary Science, Hyderabad, PVNR Telangana Veterinary University |
|  |  |  |  | West Bengal University of Animal and Fishery Sciences |
|  |  |  |  | Sher-e-Kashmir University of Agricultural Sciences and Technology |
|  |  |  |  | College of Veterinary Science and Animal Husbandry, Jabalpur |
|  |  |  |  | Veterinary College, Anand, Kamdhenu University |
|  |  |  |  | Veterinary College and Research Institute, Namakkal, Tamil Nadu Veterinary and Animal Sciences University |
|  |  |  |  | Odisha University of Agriculture and Technology (OUAT), Bhubaneswar, Odisha |
|  |  |  |  | Rajasthan University of Veterinary and Animal Sciences (RAJUVAS), Bikaner, Rajasthan |
|  |  |  |  | Rajiv Gandhi Institute of Veterinary Education and Research (RIVER), Puducherry, Puducherry |
|  |  |  |  | Govind Ballabh Pant University of Agriculture and Technology (GBPUAT), Pantnagar, Uttarakhand |
| Nigeria | ≈ 242 million (2023 population estimate) | 16 | 8 | University of Ilorin, Ilorin |
|  |  |  |  | University of Ibadan, Ibadan |
|  |  |  |  | University of Nigeria, Nsukka. |
|  |  |  |  | Usmanu Danfodiyo University, Sokoto |
|  |  |  |  | University of Maiduguri, Maiduguri |
|  |  |  |  | Michael Okpara University of Agriculture, Umudike |
|  |  |  |  | Federal University of Agriculture, Abeokuta |
|  |  |  |  | University of Jos, Jos |
| Pakistan | ≈ 241 million (2023 census) | 15 | 10 | University of Agriculture, Faisalabad. |
|  |  |  |  | University of Veterinary and Animal Science, Lahore |
|  |  |  |  | Bahauddin Zakariya University, Multan |
|  |  |  |  | PMAS Arid Agriculture University, Rawalpindi |
|  |  |  |  | Sindh Agriculture University, Tandojam |
|  |  |  |  | Kohat University of Science & Technology, Kohat |
|  |  |  |  | The Islamia University of Bahawalpur, Bahawalpur |
|  |  |  |  | Abdul Wali Khan University, Mardan |
|  |  |  |  | Khawaja Fareed University of Engineering and Information Technology, Rahim Yar Khan |
|  |  |  |  | Quaid-i-Azam University, Islamabad, Islamabad Capital Territory |
| Tunisia | ≈ 12 million (2023 population estimate) | 1 | 1 | École Nationale de Médecine Vétérinaire (ENMV), Sidi Thabe. |
| Turkey | ≈ 87 million (2023 population estimate) | 31 | 3 | Atatürk University, Erzurum |
|  |  |  |  | Kafkas University, Kars |
|  |  |  |  | Muğla Sıtkı Koçman University (Milas Faculty of Veterinary Medicine), Muğla |
